# Supplementary material for: Genotypic analyses and virulence characterization of Glaesserella parasuis isolates from Taiwan
Source: PeerJ. 2019 May 17;7:e6960. doi: 10.7717/peerj.6960 (PMC6526895; doi:10.7717/peerj.6960)
Supplement: Supplemental Information 1 [file peerj-07-6960-s001.docx]

Supplementary file 1 Description of pig farms included in this study

| Farm | Location† | Area | Production mode | Scale‡ | Isolate number |
| --- | --- | --- | --- | --- | --- |
| F1-1 | PT | Southern Taiwan | Nursery | 1700 | 56 |
| F1-2 | PT | Southern Taiwan | Nursery | 1400 | 100, 139 |
| F1-3 | PT | Southern Taiwan | Sow to nursery | 500 | 108 |
| F1-4 | YL | Central Taiwan | Finisher | 2000 | 112 |
| F1-5 | TN | Southern Taiwan | Nursery | 900 | 121 |
| F1-6 | KH | Southern Taiwan | Nursery | 1400 | 140 |
| F2-1 | PT | Southern Taiwan | Sow to nursery | 1500 | 10 |
| F2-2 | PT | Southern Taiwan | Sow farm | 5000 | 13, 96 |
| F2-3 | TN | Southern Taiwan | Nursery | 10000 | 18, 45, 46, 70 |
| F2-4 | PT | Southern Taiwan | Nursery | 2000 | 31, 32, 76 |
| F2-5 | PT | Southern Taiwan | Nursery | 6000 | 33 |
| F2-6 | YL | Central Taiwan | Nursery | 2200 | 119 |
| F3-1 | PT | Southern Taiwan | Farrow-to-finish | 1900 | 9 |
| F3-2 | PT | Southern Taiwan | Farrow-to-finish | 2000 | 20, 51 |
| F3-3 | PT | Southern Taiwan | Farrow-to-finish | 1700 | 43, 85 |
| F3-4 | PT | Southern Taiwan | Farrow-to-finish | 2000 | 49 |
| F3-5 | PT | Southern Taiwan | Farrow-to-finish | 1900 | 115, 116, 144 |
| F4 | PT | Southern Taiwan | Farrow-to-finish | 2000 | 44, 92 |
| F5 | PT | Southern Taiwan | Farrow-to-finish | 3000 | 1, 3, 8, 28, 37, 71, 79, 90, 97, 111, 128 |
| F6 | PT | Southern Taiwan | Farrow-to-finish | 150 | 64 |
| F7 | KH | Southern Taiwan | Farrow-to-finish | 400 | 134, 135 |
| F8 | PT | Southern Taiwan | Farrow-to-finish | 300 | 93 |
| F9 | PT | Southern Taiwan | Farrow-to-finish | 800 | 124 |
| F10 | CW | Central Taiwan | Farrow-to-finish | 1500 | 98 |
| F11 | CW | Central Taiwan | Farrow-to-finish | 500 | 42 |
| F12 | PT | Southern Taiwan | Farrow-to-finish | 500 | 82 |
| F13 | PT | Southern Taiwan | Nursery | 1000 | 4 |
| F14 | PT | Southern Taiwan | Farrow-to-finish | 400 | 7 |
| F15 | PT | Southern Taiwan | Farrow-to-finish | 100 | 11 |
| F16 | PT | Southern Taiwan | Farrow-to-finish | 150 | 34, 81 |
| F17 | PT | Southern Taiwan | Sow to nursery | 150 | 29 |
| F18 | PT | Southern Taiwan | Sow to nursery | 150 | 6 |
| F19 | PT | Southern Taiwan | Farrow-to-finish | 100 | 60 |
| F20 | YL | Central Taiwan | Farrow-to-finish | 130 | 22, 87 |
| F21 | YL | Central Taiwan | Farrow-to-finish | 700 | 129 |
| F22 | PT | Southern Taiwan | Farrow-to-finish | 120 | 41 |
| F23 | PT | Southern Taiwan | Farrow-to-finish | 70 | 36 |
| F24 | YL | Central Taiwan | Farrow-to-finish | 300 | 118 |
| F25 | PT | Southern Taiwan | Farrow-to-finish | 250 | 120 |
| F26 | PT | Southern Taiwan | Farrow-to-finish | 500 | 114 |
| F27 | PT | Southern Taiwan | Farrow-to-finish | 280 | 94 |
| F28 | TN | Southern Taiwan | Farrow-to-finish | 700 | 12 |
| F29 | KH | Southern Taiwan | Farrow-to-finish | 400 | 117 |
| F30 | TN | Southern Taiwan | Farrow-to-finish | 200 | 113 |
| F31 | PT | Southern Taiwan | Finisher | 1800 | 86 |
| F32 | PT | Southern Taiwan | Farrow-to-finish | 800 | 69 |
| F33 | PT | Southern Taiwan | Farrow-to-finish | 300 | 19 |
| F34 | PT | Southern Taiwan | Farrow-to-finish | 1700 | 48, 57, 137 |
| F35 | PT | Southern Taiwan | Sow to nursery | 1000 | 126 |
| F36 | TN | Southern Taiwan | Farrow-to-finish | 120 | 14, 52 |
| F37 | YL | Central Taiwan | Farrow-to-finish | 100 | 125 |
| F38 | YL | Central Taiwan | Farrow-to-finish | 250 | 75 |
| F39 | PT | Southern Taiwan | Sow farm | 500 | 127 |
| F40 | PT | Southern Taiwan | Farrow-to-finish | 100 | 65, 66, 67 |
| F41 | PT | Southern Taiwan | Farrow-to-finish | 100 | 17 |
| F42 | PT | Southern Taiwan | Nursery | 1000 | 80 |
| F43 | PT | Southern Taiwan | Farrow-to-finish | 400 | 68 |
| F44 | PT | Southern Taiwan | Nursery | 2000 | 58 |
| F45 | HL | Eastern Taiwan | Farrow-to-finish | 100 | 145 |
| F46 | PT | Southern Taiwan | Farrow-to-finish | 300 | 23 |
| F47 | PT | Southern Taiwan | Farrow-to-finish | 250 | 141 |
| F48 | PT | Southern Taiwan | Sow to nursery | 30 | 27 |
| F49 | PT | Southern Taiwan | Nursery | 1700 | 105 |
| F50 | PT | Southern Taiwan | Nursery to finish | 10000 | 104, 109 |
| F51 | TN | Southern Taiwan | Farrow-to-finish | 500 | 16 |
| F52 | PT | Southern Taiwan | Farrow-to-finish | 250 | 102, 103 |
| F53 | PT | Southern Taiwan | Sow to nursery | 800 | 62, 77, 78 |
| F54 | PT | Southern Taiwan | Farrow-to-finish | 200 | 83, 84 |
| F55 | PT | Southern Taiwan | Sow to nursery | 100 | 25 |
| F56 | PT | Southern Taiwan | Unknown§ | Unknown | 15 |
| F57 | YL | Central Taiwan | Farrow-to-finish | 400 | 2, 26 |
| F58 | YL | Central Taiwan | Farrow-to-finish | 550 | 50 |
| F59 | CW | Central Taiwan | Farrow-to-finish | 200 | 132, 133 |
| F60 | KH | Southern Taiwan | Farrow-to-finish | 120 | 53 |
| F61 | PT | Southern Taiwan | Sow farm | 180 | 47 |
| F62 | PT | Southern Taiwan | Farrow-to-finish | 1300 | 72, 73, 74, 101 |
| F63 | PT | Southern Taiwan | Sow to nursery | 100 | 106, 107 |
| F64 | KH | Southern Taiwan | Farrow-to-finish | 250 | 138 |
| F65 | PT | Southern Taiwan | Nursery to finish | 6000 | 21 |
| F66 | PT | Southern Taiwan | Sow farm | 160 | 24 |
| F67 | PT | Southern Taiwan | Farrow-to-finish | 1000 | 35, 130, 131 |
| F68 | KH | Southern Taiwan | Farrow-to-finish | 300 | 63 |
| F69 | PT | Southern Taiwan | Sow to nursery | 70 | 40 |
| F70 | PT | Southern Taiwan | Farrow-to-finish | 1100 | 136, 143 |
| F71 | PT | Southern Taiwan | Sow to nursery | 120 | 61 |
| F72 | PT | Southern Taiwan | Sow to nursery | 60 | 59 |
| F73 | PT | Southern Taiwan | Nursery to finish | 800 | 39 |
| F74 | PT | Southern Taiwan | Sow to nursery | 170 | 91 |
| F75 | KH | Southern Taiwan | Farrow-to-finish | 250 | 54, 89, 99, 110, 142 |
| F76 | PT | Southern Taiwan | Farrow-to-finish | 150 | 30 |
| F77 | KH | Southern Taiwan | Farrow-to-finish | 200 | 55 |
| F78 | PT | Southern Taiwan | Finisher | 1500 | 5 |
| F79 | KH | Southern Taiwan | Farrow-to-finish | 250 | 38 |
| F80 | PT | Southern Taiwan | Farrow-to-finish | 250 | 122 |
| F81 | PT | Southern Taiwan | Sow to nursery | 150 | 88 |
| F82 | PT | Southern Taiwan | Farrow-to-finish | 200 | 95 |
| F83 | PT | Southern Taiwan | Sow farm | 200 | 123 |

†County name abbreviations: Changhua (CW), Yunlin (YL), Tainan (TN), Kaohsiung (KH), Pingtung (PT), Hualien (HL).

‡Scales of Sow, Sow to nursery and farrow-to-finish farms were only calculated number of sows.

§Data was unknown.
